# Supposed Case of Inflammation of the Pancreas
*Med. Chir. Trans. Vol. XVI. Part I.


**Published:** 1831

**Authors:** 


### XXXII.

### SUPPOSED CASE OF INFLAMMATION OF THE PANCREAS.*

IF little is known respecting the morbid affections and conditions of the pancreas, it is partly because the gland, like the other salivary glands, is little prone to disease; and partly because its situation and other circumstances have conspired to render examinations of its state after death comparatively unfrequent. Inflammation of the pancreas has not been described by Baillie, Meckel or Andral. Portal pronounces it of common occurrence, but probably the assertion is not founded on satisfactory grounds. The following case is looked upon by Mr. Lawrence as an instance of the affection in question. Mr. L. was not requested to see the patient until thirty-six hours before her death; she was attended during the progress of the disease by a very intelligent general practitioner and two physicians. The history of the case is given in the words of the former, nor do we see that it admits of abridgement.

*Case.* "At the time Mrs. —— married, she appeared to be in good health. When she was between five and six months advanced in pregnancy, she lost her usual healthy appearance, and gradually became very pallid. This change I observed on occasionally meeting her in her walks from her own to her mother's house, and on enquiring generally after her health, her answer invariably was, 'I am quite well.'

About a month previous to her confinement, I, for the first time, was desired to see her professionally. She was then suffering from a severe attack

* Med. Chir. Trans. Vol. XVI. Part I.

of catarrh, accompanied with an incessant, irritating cough; it was attended with very little fever, her pulse, in frequency, being but little above the natural standard. This complaint yielded in about ten days to the usual remedies, when she declared herself to be quite well, and during its continuance no symptom was complained of that was not strictly catarrhal. Her skin was then completely bleached, and the prolabia colourless. I did not see her again until the 29th of January, the morning on which her labour commenced; she then looked and felt extremely exhausted, and I was anxious as to the result of her labour. On making the usual enquiry I found the presentation natural, the pains returning at pretty regular intervals; and she was delivered of a healthy female child. The placenta was expelled by the contraction of the uterus five minutes afterwards, and she did not, during the whole labour, lose two ounces of blood. The night after her labour was passed without pain; she was tolerably tranquil, but got little sleep. It was evident on the third day after her delivery, that although the labour was comparatively easy, she had suffered much from the exertion. She felt so exhausted that she was constantly calling for sal volatile to smell, and occasionally to take internally, in order to prevent fainting: she sighed deeply and frequently. The least attempt to raise her head from the pillow produced a violent beating in the temples, but it subsided after a few minutes of perfect quietude. Her pulse was feeble and irritable, at about eighty-six beats in a minute. The bowels were rather relaxed. She was very thirsty, and had been so for three months previous to her delivery.

On the fifth day after her confinement, Dr. —— saw her, and he repeated the examination I had previously made, by pressure with the hand over the whole abdominal cavity, in order to discover if there was tenderness in any part, but our patient declared most positively, she felt neither pain nor soreness from the pressure. A similar examination was made some days afterwards with the same result. The feeling of exhaustion continued to increase, but she never complained of pain till about a week before her death, when, on pressing the abdomen, a slight uneasiness was felt about the situation of the caput coli. This was noticed the following day by Dr. ——, who directed a mustard poultice to be applied to the part. About five days previous to her death the stomach became irritable, and nothing but rennet whey in small quantities was retained. She died exactly five weeks after her delivery.

I should observe, that I do not recollect to have heard of Mrs. —— suffering severe pain in the epigastrium till it was mentioned by her mother after her death: she certainly never named it to me herself, and it does appear somewhat extraordinary that, when it existed, it should not have been thought of sufficient consequence to call for medical assistance."

We should observe, that the mother of the lady stated that she had been singularly troubled with thirst during her pregnancy, that the pain was occasionally very severe, and referred exactly to the situation of the pancreas.

*Sectio Cadaveris, thirteen hours after death.* The internal parts of the body were still warm, the external parts blanched. The membranes, the thorax, and abdomen, and the viscera, with the exception of the spleen and pancreas were pale and almost bloodless. The cellular texture round the pancreas and duodenum, the omenta, the root of the mesentery, the mesocolon and appendices epiploical of the arch of the colon were loaded with serous effusion. The pancreas was throughout of a deep and dull red colour, firm to the feel externally, and when divided the lobules felt firm and crisp. After the gland had been wrapped in a cloth for forty-eight hours (the weather being very cold) the hardness disappeared. There was some serous effusion into the pia mater of the brain, the vessels of which were moderately full.

We know not whether the case was satisfactory to Mr. Lawrence; it certainly is not so to us. Granting that the appearances discovered after death were really produced by inflammation of the pancreas, still that inflammation had not proceeded to disorganization of the gland, to suppuration, ulceration, nor even to consolidation; nor is there, on the face of the report, any evidence to prove that its secretion was not and could not be performed. So far as we know, the pancreas is not absolutely essential to life; at all events, chronic inflammation of its structure which left no trace save redness behind it, can hardly be thought capable of producing death. We cannot look on the case as satisfactory, for the existence of no other morbid alterations of consequence throws little light on the enquiry. At the same time the profession will feel obliged to Mr. Lawrence for publishing the particulars.

---

